# Sensor Effects in LCL-Type Grid-Connected Shunt Active Filters Control Using Higher-Order Sliding Mode Control Techniques

**DOI:** 10.3390/s22197516

**Published:** 2022-10-03

**Authors:** Mohamad Alaa Eddin Alali, Yuri B Shtessel, Jean-Pierre Barbot, Stefano Di Gennaro

**Affiliations:** 1 QUARTZ Laboratory, EA7393, ENSEA, 6 Avenue du Ponceau, 95014 Cergy-Pontoise, France; 2 Department of Electrical and Computer Engineering, University of Alabama in Huntsville, 301 Sparkman Drive, Huntsville, AL 35801, USA; 3 Laboratoire des Sciences du Numérique de Nantes, Nantes University-Ecole Centrale de Nantes-LS2N, UMR 6004 CNRS, 44307 Nantes, France; 4 Department of Information Engineering, Computer Science and Mathematics, Center of Excellence DEWS, University of L’Aquila, Via Vetoio, Loc. Coppito, 67100 L’Aquila, Italy

**Keywords:** shunt active filters, higher-order sliding mode control, advanced PLL, power quality analyzer, current and voltage sensors

## Abstract

The effects of measuring devices/sensors on improving the power quality (PQ) of electric networks are studied in this paper. In this context, improving the performance of an LCL-type grid connected to a three-phase three-wire shunt active filter (SAF) in the presence of voltage perturbations is studied. In order to ensure the high-quality performance of LCL-SAF in the presence of voltage perturbations, the robust continuous second-order sliding mode controller (2-SMC), including twisting and super-twisting controllers, and continuous higher-order sliding mode controller (C-HOSMC)-based approaches are employed. These controllers, whose outputs are processed by pulse-width modulation (PWM), allow minimization of the phase shift and prevent the generation of discontinuous chattering commands, which can severely damage the VSI components. Moreover, an integration of a generalized instantaneous power identification algorithm with an advanced phase locked loop (PLL) was proposed and experimentally tested to validate the effective performances of SAF under severe perturbations. Additionally, the studied approaches were tested via simulations taking into account a conventional nonlinear industrial load in a real textile factory environment, using measurements provided by power quality analyzers. Finally, the effects of the measuring devices, including the current and voltage sensors, on the accuracy and reliability of the SAF and, consequently, on the PQ of the electric power grid were studied via simulations and experimentally. The results of this study support the validity of the recently published patent.

## 1. Introduction

The efficacy and sustainability of power electronics play a fundamental role in power systems, where voltage source inverters (VSIs) are used for energy conversion processes, both in standalone and in grid-connected inverters.

Filters, such as an L-series inductor (first-order coupling/output filter), are used to connect VSI to the grid while attenuating the switching components generated by VSIs [1]. The L-series/VSI structure requires heavy, bulky, expensive, and difficult to size output inductances [1]. However, LC, LCL, and LCLCL output filters provide, in comparison with first-order filters, higher attenuation of high-frequency components and a weight and size reduction of the passive components [2,3,4,5].

In many applications, LCL filters are used mostly for their low cost and efficient high-frequency component attenuation capabilities compared to other output filters, including L-series filters [6,7,8,9]. Therefore, LCL-type grid-connected VSIs are often employed in renewable energy systems (photovoltaic, wind, etc.) [6,10,11,12]. The role of VSIs is, in this case, to generate fundamental components (active, reactive, etc.) for the grid. 

On the other hand, current perturbations in power grids, including harmonic, unbalanced, and reactive currents, have been compensated by controllers using shunt active filters [13,14,15]. In these control systems, the current control loop ensures an accurate tracking over the entire frequency bandwidth while guaranteeing a good filtering quality of the perturbations. Specifically, a roots locus-based linear controller (RST) in concert with PWM-VSI is proposed in [3,16]. Note that while the current tracking error achieved in the current control loop is small enough, the observed phase lag between the command and measured currents may significantly reduce the filtering quality. Thus, either this controller or all the conventional linear controllers limit the shunt active compensation capability for harmonic filtration [16].

In order to address these problems, classical two-level VSIs equipped with an LCL filter and controlled by appropriate nonlinear controllers, including SMC and the neural network controller, were studied in [17,18]. The proposed algorithms provide a good attenuation of the matched disturbances [19]. Note that the high-frequency switching control function, generated by SMC, may severly damage the power devices of VSI [20]. SMC, approximated by a sigmoid function or transformed by an artificial increase in the relative degree followed by an integrator (AIRD), provide continuity to the control signal [18]. Note that the sigmoid function approximation method does not guarantee insensitivity to the matched disturbances experienced by SMC while the robust AIRD method is a more practical implementation. 

High-accuracy tracking and, consequently, good performances of the three-phase AC/DC boost converter are achieved by the higher-order sliding mode controller (HOSMC) proposed in [21]. Note that this controller does not perform well in the presence of high-frequency switching disturbances, which could severely deteriorate the filtration quality of SAF.

Moreover, the errors produced by sensors, affect the accuracy of the perturbation identification and significantly limit the power quality improvement in electric networks [22,23,24]. It is worth noting that this issue has not been fully addressed in the literature [25].

In this work, the effects of measuring device sensors on the quality of the perturbation compensation of the shunt active filter are studied. In this context, the continuous 2-SMC and C-HOSMC algorithms processed by PWM, which ensure a predefined switching frequency of LCL grid-connected VSI, are employed. While 2-SMC/C-HOSMCs maintain the robustness of SMC, no overheating of VSI switching devices is expected. Specifically, three different robust control algorithms are explored, namely: the C-HOSMC, 2-SMC twisting controller, and 2-SMC super-twisting controller. The performances of the LCL system controlled by the discontinuous SMC and the three studied 2-SMC/HOSMCs with PWM are validated and compared via simulations, which are performed for a distorted and highly distorted level of the network voltage using the MATLAB, Simulink, and Simscape-Electrical codes.

The novelty/contributions of this research are:

1. The performance in the studied LCL grid-connected SAF structure is improved, and the required switching frequency is ensured by controlling the studied structure by continuous 2-SMCs super-twisting and twisting and C-HOSMC processing by PWM. This approach prevents overheating of the inverter components and, at the same time, retains the robustness accuracy while improving the effectiveness of the entire LCL grid-connected SAF system;
2. An advanced-phase locked loop (PLL)-based generalized instantaneous power identification method is proposed and validated via an experimental study using current and voltage sensors. An appropriate sensor ensures accurate and reliable results;
3. A new approach to modeling industrial sites using real measurements provided by the sensor of the power quality analyzer, including adapted current sensors and QualyStar View software, is proposed and studied. This approach allows an improvement in the accuracy and credibility of the simulation results compared to the experimental ones.

Note that this work is based on a recently published patent [25].

## 2. Shunt Active Filter Environment and Control Problem Formulation

### 2.1. General Basic Structure

In electrical grids, the perturbations, including the current and voltage harmonics, unbalanced voltages and currents, and reactive power, represent the main concerns in terms of power quality. Therefore, shunt active filters are usually employed to eliminate the current perturbations and subsequently the voltage perturbations of the same kind [13,14,15,16,26].

**Figure 1:**
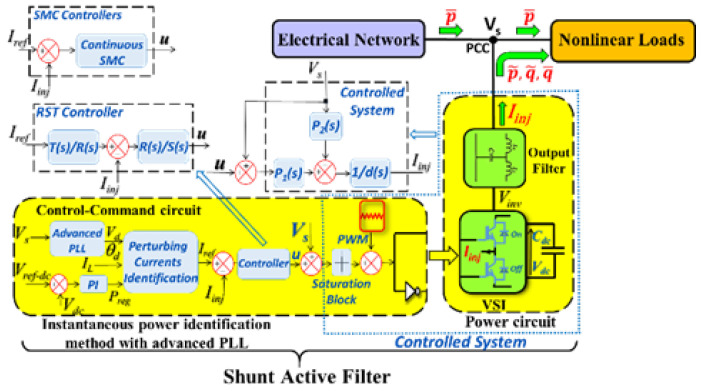
The shunt active filter environment.

A generic schematic of SAF connected in parallel between a three-wire network and a nonlinear load is shown in Figure 1, where $\tilde{p}$, $\tilde{q}$, $\bar{q}\;$represent the identified perturbing real and imaginary powers and the reactive power, respectively. The shunt active filter is generally composed of a power part and a control part, including a PWM-VSI, DC capacitive storage element, and a coupling/output passive filter. The control part consists of a current perturbation identification algorithm and control loops for injecting currents into the grid and for DC voltage regulation of the capacitive storage element.

### 2.2. LCL Filter Modeling: Transfer Function and State Space Representations

**Figure 2:**
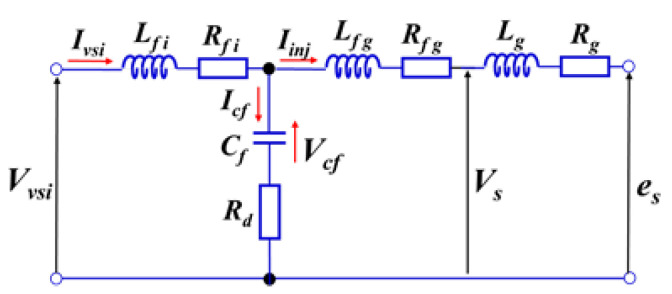
A single-phase circuit of the LCL output filter.

A PWM three-phase voltage source inverter (VSI) is used to generate the current that is injected into the grid. A passive coupling/output filter is employed to connect VSI to the grid. The output filter is designed to prevent the switching frequency components of the PWM-VSI from passing into the grid. Figure 2 shows a single-phase equivalent circuit of the LCL output filter [27], where $L_{fi}$ and $L_{fg}\;$are the inductances on the inverter and grid side, respectively, with their internal resistances $R_{fi}$ and $R_{fg}$; the capacitance $C_{f}$ is in series with the damping resistance$\;R_{d}$; and $L_{g}$ and $R_{g}$ are the equivalent inductance and resistance of the grid, respectively.

The model of the LCL filter is firstly presented in the Laplace variable (s)-domain (see [18] for more details):

(1) $$I_{inj}\left(s\right)=\left(\frac{P_{1}\left(s\right)}{d\left(s\right)}\right)\;V_{vsi}\left(s\right)+\left(\frac{P_{2}\left(s\right)}{d\left(s\right)}\right)\;V_{s}\left(s\right)$$

where the term P_1_(s)/d(s) represents the LCL transfer function, the term P_2_(s)/d(s) represents a perturbation model, $V_{vsi}\left(s\right)\;$is the inverter output voltage, and $V_{s}\left(s\right)$ is the PCC grid voltage, which, in this case, represents the disturbance.

A simplified LCL transfer function, where all internal resistances are neglected, is derived based on Figure 2 and Equation (1) as follows:

(2) $$\frac{P_{1}\left(s\right)}{d\left(s\right)}=\frac{\left(R_{d}\;C_{f}\right)s+1}{\left(L_{fi}\;L_{fg}\;C_{f}\right)s^{3}+R_{d}\;C_{f}\left(L_{fi}+L_{fg}\right)s^{2}+\left(L_{fi}+L_{fg}\right)s}$$

It is worth noting that:

(a) In electrical grids, all disturbances $\left(\frac{P_{2}\left(s\right)}{d\left(s\right)}\right)V_{s}\left(s\right)$ are bounded, which implies that $V_{s}\left(s\right)$ is bounded and d(s) is Hurwitz (see [18] for details).
(b) Usually, in industrial applications, passive damping is not permitted for active power consumption reasons.

With the computed cut-off frequency:

(3) $$f_{c-off}=\frac{1}{2\pi \sqrt{\left(\frac{L_{fi}\;L_{fg}\;C_{f}}{L_{fi}+L_{fg}}\right)}}$$

The state space model of the single-phase LCL output filter is derived [18] based on Equation (1):

(4) $$\dot{x}=A\;x+B\;v_{VSI}+P\;v_{s}$$

where$\;x=\left[\begin{array}{c} i_{vsi}\\ i_{inj}\\ v_{cf} \end{array}\right]$ is a state vector, $i_{inj}$ is the grid-injected current, $v_{cf}\;$is the capacitor voltage, $i_{vsi}$ is the inverter side current, $v_{cf}$ is the capacitor voltage, $u=v_{vsi}$ is a control function, and $v_{s}$ is the bounded disturbance, and:

$$A=\left[\begin{array}{ccc} -\frac{R_{fi}+R_{d}}{L_{fi}} & \frac{R_{d}}{L_{fi}} & -\frac{1}{L_{fi}}\\ \frac{R_{d}}{L_{fg}} & -\frac{R_{fg}+R_{d}}{L_{fg}} & \frac{1}{L_{fg}}\\ \frac{1}{C_{f}} & -\frac{1}{C_{f}} & 0 \end{array}\right],\;B=\left[\begin{array}{c} \frac{1}{L_{fi}}\\ 0\\ 0 \end{array}\right],\;P=\left[\begin{array}{c} 0\\ -\frac{1}{L_{fg}}\\ 0 \end{array}\right]$$

### 2.3. Current Control Objective

The control objective of the current tracking loop of SAF is, among others, to ensure a high level of filtering quality over the entire bandwidth harmonic frequencies, including the fundamental one. Thus, the controller must provide asymptotic convergence of the tracking error, i.e., $e_{i}\left(t\right)=i_{ref}\left(t\right)-i_{inj}\left(t\right)\to 0$ as time increases, to zero in the amplitude and the phase. Therefore, the control problem consists of designing the continuous control $u=v_{inv}$ so that $\underset{t\to \infty }{\lim}\left(i_{ref}-i_{inj}\right)=0$ in the presence of the bounded disturbance $v_{s}:\left|v_{s}\right|\leq L,\;L>0$ with a consecutive PWM that enforces the desired switching frequency of the inverter.

## 3. State of the Art of LCL/SAF Structure Controllers

Two control loops, in addition to other algorithms, are necessary to ensure a good performance of the shunt active filter. These are the current controller and the DC bus voltage controller.

In this section, the previously proposed and studied linear (RST) and nonlinear controllers (SMC) for the LCL-type grid-connected SAF are reviewed. The advantages and disadvantages of these control algorithms employed for harmonic filtration purposes are discussed. In addition, the closed-loop regulation of DC voltage is also presented and detailed.

### 3.1. Grid-Connected SAF with an LCL Filter Controlled by the Linear RST Approach 

The control loop is designed to ensure high-accuracy tracking $i_{inj}\to \;i_{ref}$ as time increases. The general block scheme of the SAF’ current control loop is shown in Figure 1. Specifically, the control-command part of Figure 1 depicts the LCL grid-connected SAF with the root locus (RST) controller and the instantaneous real and imaginary power algorithm for identifying the current perturbation [16,26]. The advanced-phase locked loop (PLL) provides both the magnitude $V_{d}\;$and the phase $\theta_{d}$ and consequently the positive sequence voltage components (PSVCs) $V_{d123}$ of the grid voltage $V_{s}$ [16]. Thus, an accurate current identification is guaranteed even under an unbalanced and distorted grid [25].

Next, the DC voltage control loop of the capacitive storage element is used to provide a fixed and appropriate voltage on the DC side inverter.

### 3.2. Advanced PLL-Based Generalized Current Identification Algorithm within the LCL/SAF Structure Controllers 

**Figure 3:**
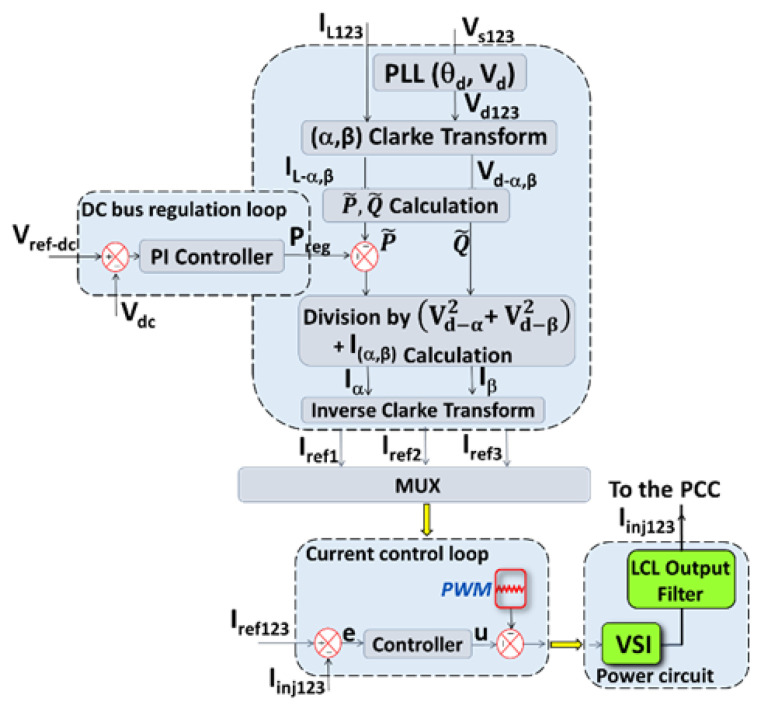
Advanced PLL-based generalized instantaneous power algorithm.

Figure 3 shows the overall control-command scheme of the LCL/SAF environment, including the generalized current identification algorithm with the advanced PLL system.

The generalized method for identifying/computing the reference currents includes, as shown in Figure 3, the following steps:

1. Processing of the measurements of the load current I_L123_ and the grid voltage V_s123_ is carried out in order to estimate the harmonic, reactive, unbalanced, etc. currents. A loop for regulating the voltage of the capacitor of VSI is used to charge the capacitor of the inverter in order to compensate for the losses caused by the components of the inverter and the LCL filter.
2. The output of the DC regulation voltage controller, i.e., the regulating power P_reg_, is added to the instantaneous active disturbance power $\tilde{p}$.

Then, the generalized identification algorithm divides the instantaneous power by the square module of the (PSVC) $V_{d123}$ in (α, β) coordinates. This operation ensures an accurate extraction of the perturbing current under highly distorted networks conditions while avoiding consumption of the additional active current from the grid to charge the capacitor $C_{dc}$. Indeed, the use of the classical PLL, which only extracts the phase $\theta_{d}$ by dividing the instantaneous real and imaginary powers by a voltage amplitude lower than $V_{d}$, results in additional active current consumption from the electrical network.

Furthermore, this algorithm incorporates a very simple DC voltage regulation loop while ensuring, using the advanced PLL, the DC bus charging current is in phase with the PCC voltage, preventing additional reactive power consumption.

#### 3.2.1. DC Voltage Regulation

For the DC bus voltage control, the use of a proportional-integral controller, as shown in Figure 1, is proposed. Then, neglecting the switching losses in the inverter, the relation between the regulating power $P_{reg}\;$and the capacitor voltage $C_{dc}$ can be written as follows [28]:

(5) $$P_{reg}=\frac{d}{dt}\left(\frac{1}{2}C_{dc}\times V_{dc}^{2}\right)\;\;$$

Then, assuming small variations in the DC bus voltage $V_{dc}$, Equation (5) is linearized in the vicinity of the reference voltage as $V_{ref-dc}$: $P_{reg}=C_{dc}V_{ref-dc}\frac{d}{dt}\left(V_{dc}\right)$.

Finally, the DC voltage is given in the Laplace variable domain:

(6) $$V_{dc}\left(s\right)=\frac{P_{reg}\left(s\right)}{V_{ref-dc}\;C_{dc}\;s}$$

In this work, the DC bus capacitance is computed as in [3,7]:

(7) $$C_{dc}=\frac{\;I_{h}}{\varepsilon \;V_{dc}\;\omega_{h}}$$

where $I_{h}$ is the current harmonic of the lowest rank, $\omega_{h}$ is the pulse, and ε is an acceptable ripple rate.

In this context, it is important to mention that voltage fluctuations lead to incorrect operation of powered loads and could severely affect the voltage distortion caused by power electronic loads [27,29]. In this article, under voltage fluctuation conditions caused by sub-harmonics, inter-harmonics (other than the flicker aspect) and high-frequency switching power electronic-based loads, the advanced PLL extracts the direct component grid voltage, ensuring the identification and then the filtration of all the sub-harmonics/inter-harmonics/high harmonics current orders and, consequently, the relating voltage distortion/fluctuation, such as in the case of conventional harmonics. It is worth noting that flicker perturbations, caused, for example, by iron and steel plants, must be treated by the series active filters or UPQC (unified power quality conditioner) [30].

#### 3.2.2. Advanced-Phase Locked Loop

On the basis of the three-phase PCC voltage ($v_{s1},\;v_{s2},\;v_{s2}$), the $V_{sd}$ and $V_{sq}$ components are calculated in the Park representation using the estimated rotation angle of ${\hat{\theta }}_{d}$; $\theta_{d}=\omega_{d}t+\delta_{d}$, where $\omega_{d}$ and $\delta_{d}$ represent the pulse and the phase of PSVC, respectively. 

Finally, in the d−q coordinates, we obtain:

(8) $$\left(\begin{array}{c} V_{sd}\\ V_{sq} \end{array}\right)={\left[C_{32}\right]}^{t}\cdot P\left(-{\hat{\theta }}_{d}\right)\cdot v_{s123}\left(\theta_{d}\right)=\sqrt{3}V_{d}\left[\begin{array}{c} \sin\left(\theta_{d}-{\hat{\theta }}_{d}\right)\\ -\cos\left(\theta_{d}-{\hat{\theta }}_{d}\right) \end{array}\right]\;\approx \sqrt{3}V_{d}\left[\begin{array}{c} \sin\left(\Delta \theta_{d}\right)\\ -\cos\left(\Delta \theta_{d}\right) \end{array}\right]$$

where $V_{d}$ is the amplitude of PSVC.

The phase lock occurs when $\Delta \theta_{d}=0$. In this case, the actual and estimated angles of PSVC are equal, i.e.,

(9) $$V_{sd}=0\;\mathrm{and}\;V_{sq}=-\sqrt{3}V_{d}$$

It is worth noting that phase and amplitude tracking are ensured using an adapted RST controller, where R(s), S(s), and T(s) are the controller polynomials of the Laplace variable s. This controller has to provide a robust performance over wide frequency and voltage variations. In addition, it should filter the inner disturbances (caused by unbalanced and harmonic components) in the closed loop. For these purposes, the fifth- and sixth-order polynomials R(s) and S(s) are selected, respectively, while T(s) is the gain that provides $\frac{V_{ref-d}}{V_{d}}=1$ [25].

#### 3.2.3. Current Root Locus Current Controller Design

Imbedding the RST controller, given in [16,18], in system (1) yields the following closed-loop system dynamics in the Laplace domain:

(10) $$I_{inj}\left(s\right)=\frac{\left(T\left(s\right)P_{1}\left(s\right)\right)}{\left(S\left(s\right)d\left(s\right)+R\left(s\right)P_{1}\left(s\right)\right)}I_{ref}\left(s\right)+\frac{\left(S\left(s\right)P_{2}\left(s\right)\right)}{\left(S\left(s\right)d\left(s\right)+R\left(s\right)P_{1}\left(s\right)\right)}V_{s}\left(s\right)$$

The two parts of Equation (10) represent the closed-loop transfer function in terms of the tracking control and disturbance compensation. For continuous time representations, the third-order R(s) and S(s) polynomials are chosen in the LCL filter: $P_{1}\left(s\right)/d\left(s\right)$ [16,18]. The gain T(s) is chosen to ensure $(I_{ref}/I_{inj})\left(s\right)=1$ for the entire bandwidth harmonic frequencies. It is worth noting that the transfer function of the controller is represented by R(s)/S(s) (see Figure 1) and the common denominator closed-loop characteristic polynomial $D\left(s\right)=\left(S\left(s\right)d\left(s\right)+R\left(s\right)P_{1}\left(s\right)\right)$. Its roots are the poles of the closed loop. These poles, whose locus is in a sector (2×45°), provide a damping rate of 0.7. Moreover, the closed-loop root locus ensures accurate and fast tracking while providing a complete perturbation rejection [16]. It should be noted that the location of the roots of the characteristic polynomial are constrained by the cutoff frequency of the closed loop at the tracking level.

#### 3.2.4. Phase Shift Impact of Linear Controllers

The RST controller is usually employed when the reference current is a slow varying (within a bandwidth of the closed-loop system) signal. In this case, the phase shift between the reference $i_{ref}$ and injected $i_{inj}$ currents in the low single-frequency case (i.e., reactive power consumption and/or unbalance enhancement) can be neglected. However, the phase shift has to be taken into account if the reference current contains high-frequency terms that are beyond the bandwidth of the closed-loop system. In this case, the higher the output filter order, the larger the phase shift. Then, the phase lag effect prevents the applicability of the LCL/SAF structure associated with RST as well as all conventional linear controllers [16,18].

### 3.3. LCL/SAF Current Control: Sliding Mode Control Approach 

#### 3.3.1. State of the Art and Problem Statement

The grid-connected VSI with an LCL filter is usually driven by control laws, originally proposed for renewable energy (photovoltaic, wind, hydrogen fuel cell, etc.)-based converters [4,8,9,11]. A variety of linear control laws for this kind of power converter, whereby only components at a fundamental frequency are injected into the network, have been proposed and studied, for instance, in [13,31,32,33].

However, in the case of SAF, the generated voltage may yield and inject both fundamental (unbalanced and reactive) and harmonic currents. Then, the source inverter has to have full control of the entire bandwidth frequencies. It is worth noting that the large phase lag naturally limits the use of linear controllers in the case of harmonic filtering.

A sliding mode controller (SMC) was proposed [2,8,18] in order to address these problems while providing rejection of the matched disturbances and ensuring a high tracking accuracy of$\;i_{inj}\left(t\right)\to \;i_{ref}\left(t\right)$ as time increases in the gain and phase and, consequently, a high filtering quality.

#### 3.3.2. SMC Design 

The system (2) is considered, where $y∶=i_{inj}\;$is the output, $u∶=v_{inv}$ is the control input, and $w∶=v_{s}$ is the bounded perturbation, and the damping resistance $R_{d}=0$.

Then, Equation (2) becomes:

(11) $$\dot{x}=Ax+Bu+Pw,\;y=Cx,C=\left[0\;1\;0\right]$$

where:

$$A=\left[\begin{array}{ccc} -\frac{R_{fi}}{L_{fi}} & 0 & -\frac{1}{L_{fi}}\\ 0 & -\frac{R_{fg}}{L_{fg}} & \frac{1}{L_{fg}}\\ \frac{1}{C_{f}} & -\frac{1}{C_{f}} & 0 \end{array}\right],\;B=\left[\begin{array}{c} \frac{1}{L_{fi}}\\ 0\\ 0 \end{array}\right],\;P=\left[\begin{array}{c} 0\\ -\frac{1}{L_{fg}}\\ 0 \end{array}\right]\;$$

It is easy to show that the input-output (y,u) dynamics of system (11) have a relative degree r=3, since CB=CAB=0 and $CA^{2}B=\frac{1}{\left(L_{fi}L_{fg}C_{f}\right)}$.

Therefore, a sliding variable can be chosen as [33]:

(12) $$\sigma =\beta_{0}e+\beta_{1}\dot{e}+\beta_{2}\ddot{e}$$

where $e=i_{inj}-i_{ref}$ (Cf. Figure 3). Note that the derivatives $\dot{e},\;\ddot{e}$ may be obtained using the higher-order sliding mode differentiator [34]. The positive coefficients $\beta_{0},\beta_{1}$, and $\beta_{2}$ are selected to make the system (11) exponentially stable with the desired convergence rate in the sliding mode defined by σ=0.

In order to design SMC that drives σ→0 in finite time and keeps the states of the system (11) in the sliding surface σ=0 for all consecutive time, the sliding variable dynamics are derived (see [18] for more details).

The sliding mode existence condition [19] $\sigma .\;\dot{\sigma }\;<0$ can easily be reached by SMC:

(13) $$u∶=v_{inv}=-\lambda \;sign\;\left(\sigma \right)$$

where the gain λ is set to 420 V, which represents the saturation limiter block (see Figure 1).

Note that the SMC algorithm robustly provides high accuracy tracking with a finite time convergence. The states of the system converge to the sliding surface in the presence of the bounded perturbations. However, it generates very-high-frequency switching control that could lead to overheating and consequently damage the power electronic devices of the inverter [18,20].

In this work, a variety of continuous 2-SMC/C-HOSMC, namely the C-HOSM, 2-SMC twisting, and 2-SMC super-twisting controllers, are employed in order to guarantee a good filtering quality of SAF, ensured by SMC but with continuous control functions. It is shown that these controllers provide a desired system’s dynamics in the sliding mode while guaranteeing a strong robustness to the matched bounded disturbances and enhanced stabilization accuracy in a wide range of operating conditions [19]. Furthermore, unlike classical SMC, 2-SMC and C-HOSMC can handle the sliding variable dynamics with a higher order (higher relative degree), which allows more flexibility in the sliding variable design.

## 4. Main Results: LCL/SAF Current Control: Second- and Higher-Order Sliding Mode Approaches

### 4.1. C-HOSMC Design

Note that the C-HOSMC algorithm [34,35,36] can handle systems with an arbitrary relative degree. Here, the sliding variable is designed as:

(14) $$\sigma =\dot{e}+c\;e$$

And it has a relative degree r = 2 in accordance with Equation (11). 

The rationale behind Equation (14) is as follows. In a noisy measurement environment, the sliding variable σ will converge to a domain, whose size is proportional to the amplitude of the noise w(t) of the σ measurement. Therefore, in the real sliding mode, the sliding variable dynamics are defined as:

(15) $$\sigma =\dot{e}+c\;e=w\left(t\right)$$

And the noise effect on the tracking error e is attenuated due to low pass filtering with a cut-off frequency equal to c (in our case, $c=10^{4}$).

The sliding variable σ input-output dynamics are derived in accordance with Equations (11) and (14):

(16) $$\ddot{\sigma }=v+f\left(x,t\right)$$

where $v=\frac{1}{\left(\left(L_{g}+L_{fg}\right)\;L_{fi}\;C_{f}\right)}u$, where u is the controller output (see Figure 1) and f(x,t), representing the commutation perturbation term, as the derivative of the term f(x,t) is assumed to be bounded at least locally, i.e.,$\;\left|\dot{f}\left(x,t\right)\right|\leq L$, L>0.

Note that STW control cannot directly handle the sliding variable (16) of relative degree 2. This is why C-HOSMC is applied. 

In accordance with the C-HOSMC algorithm [34,35], the control law is designed as:

(17) $$v=v_{1}-v_{2}$$

where:

(18) $$v_{1}=-\gamma_{1}{\left\lceil \sigma \right\rfloor }^{\alpha_{1}}-\gamma_{2}{\left\lceil \dot{\sigma }\right\rfloor }^{\alpha_{2}}$$

Additionally:

- $\gamma_{1},\;\gamma_{2}$ are selected to make the polynomial $p^{2}+\gamma_{2}p+\gamma_{1}$ Hurwitz with the desired root placement;
- $(\alpha_{1},\;\alpha_{2}$) are computed as $\alpha_{1}=\alpha /\left(2-\alpha \right)$, $\alpha_{2}=\alpha$, α∈(0,1); if α=1/2 is selected, then $\alpha_{1}=1/3,\;\alpha_{2}=1/2$, and $v_{2}$ is chosen for disturbance rejection purposes as:

(19) $$v_{2}=-\omega$$

where $\omega =-\lambda {\left\lceil S\right\rfloor }^{1/2}-\beta \;\int_{0}^{t}sign\left(S\right)dt$ and:

(20) $$S=\dot{\sigma }-\int_{0}^{t}v_{1}dt$$

Additionally, the coefficients λ, β>0 are chosen accordingly [34]. Note that the notation ${\left\lceil x\right\rfloor }^{\alpha }$ can be expanded as ${\left\lceil x\right\rfloor }^{\alpha }={\left|x\right|}^{\alpha }\;sign\left(x\right)$.

It is proven in [34,35] that the control law in Equations (17)–(20):

- Drives $\sigma ,\dot{\sigma }$ to zero in finite time;
- Is continuous.

Note that the control continuity and robustness are achieved here without artificially increasing the relative degree.

**Computation of**$\gamma_{1},\;\gamma_{2}$**:** They are computed [33,35] as coefficients of a second-order polynomial; the eigenvalues of this polynomial are chosen to provide a given transient response while being limited by the VSI switching frequency, enforced by the PWM dither signal.

**Computation of**λ, β**:** They are chosen [33] as follows:

(21) $$\lambda =1.5\sqrt{L},\;\beta =1.1\;L$$

In our case, L is set to $3\times 10^{18}$.

Then, the command u applied to the inverter switches is given as follows:

(22) $$u=\left(\left(L_{g}+L_{fg}\right)\;L_{fi}\;C_{f}\right)v$$

where $\left(L_{g}+L_{fg}\right)\;L_{fi}\;C_{f}=a_{1}\;$is the coefficient of $s^{3}$ of the transfer function $B_{1}\left(s\right)/A\left(s\right)$ of Equation (1) [18].

**Figure 4:**
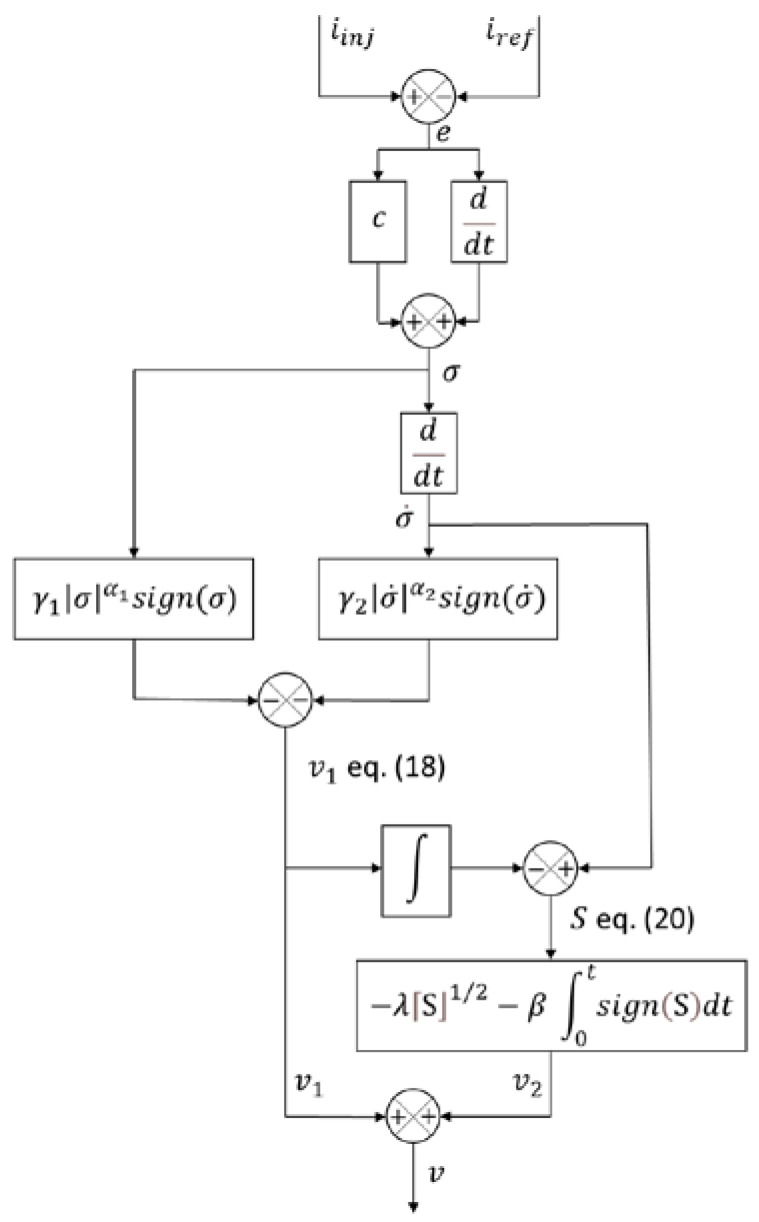
Flowchart of the C-HOSM controller.

Finally, the flowchart describing the C-HOSM controller is presented in Figure 4.

### 4.2. 2-SMC Twisting Algorithm 

The sliding variable is chosen as:

(23) $$\sigma =\gamma_{1}\;e+\gamma_{2}\;\dot{e}+\ddot{e},\;\;\;\;\gamma_{1},\;\gamma_{2}>0$$

Then, $\dot{\sigma }=v+\varphi$, where φ(x,t) represents the cumulative disturbance of the system (11).

Differentiating the derivative of the sliding variable, $\dot{\sigma }$, we obtain the sliding variable dynamics with respect to the control derivative:

(24) $$\ddot{\sigma }=\dot{v}+\dot{\varphi }$$

where $\dot{\varphi }$ is assumed to be bounded in a reasonable domain of the state variables of system (11).

The twisting control [33] that drives $\sigma ,\dot{\sigma }\;$to zero in finite time is designed in terms of the derivative of the control v:

(25) $$\dot{v}=-\alpha \left(\beta \cdot sign\left(\sigma \right)+sign\left(\dot{\sigma }\right)\right)$$

where the coefficients α, β >0 are chosen accordingly [33].

**Computation of**$\gamma_{1},\;\gamma_{2}$**:** They are computed [33,34] as the coefficients of a Hurwitz second-order polynomial; the eigenvalues of this polynomial are chosen accordingly.

**Computation of**α, β**:** They are set, respectively, to $10^{8}\;\mathrm{and}\;0.6$ in order to ensure a fast response and stability.

Note that$\;\dot{v}$ is a high-frequency switching function in the second-order sliding mode $\sigma ,\dot{\sigma }=0$ while the control:

(26) $$u=\int_{0}^{t}\dot{v}\;dt$$

is continuous and is supposed to be processed by PWM prior to application to the VSI switches.

### 4.3. 2-SMC Super-Twisting Algorithm 

The sliding variable is chosen as:

(27) $$\sigma =\gamma_{1}\;e+\gamma_{2}\;\dot{e}+\ddot{e},\;\;\gamma_{1},\;\gamma_{2}>0$$

Then:

(28) $$\dot{\sigma }=u+f\left(x,t\right)$$

where f(x,t) represents the cumulative perturbations/disturbances of the system (11), whose derivative is assumed to be bounded. The super-twisting control that drives $\sigma ,\dot{\sigma }\;$to zero in finite time is designed as [14,35,37]:

(29) $$u=-\lambda {\left\lceil \sigma \right\rfloor }^{1/2}-v$$

With:

(30) $$\dot{v}=-\beta \;Sign\left(\sigma \right)\;\mathrm{and}\;v=-\beta \;\int_{0}^{t}sign\left(\sigma \right)dt$$

where the coefficients λ, β >0 are chosen accordingly [33].

**Computation of**$\gamma_{1},\;\gamma_{2}$**:** As in the two previous cases, these parameters are computed as the coefficients of a Hurwitz second-order polynomial; the eigenvalues of this polynomial are chosen accordingly to provide a fast response while being limited by the VSI switching frequency imposed by the PWM carrier signal.

**Computation of**λ, β**:** They are chosen [34] as follows:

(31) $$\lambda =1.5\sqrt{L},\;\beta =1.1\;L$$

where L is tuned to be large enough so that $\left|\dot{f}\left(x,t\right)\right|\leq L$. L is set to $1\times 10^{7}$. Note that the super-twisting control law of Equations (29)–(31) generates a continuous control function.

## 5. Simulation Results

In this work, the filtration quality of the grid-connected LC-based SAF filter associated with the studied continuous 2-SMC and C-HOSMC are validated via two simulation setups, performed using MATLAB, Simulink, and Simscape-Electrical. The first setup study reflects a commonly used industrial nonlinear load case (modeling a UPS system) and the second one investigates a real case study. For the first simulation setup, the network is modeled by a 20/0.4 kV power distribution transformer fed by an electrical network, whose short-circuit power Ssc = 500 MVA, which is presented by its short-circuit inductance and resistance ($L_{sc},\;R_{sc}$) in series with a rated upstream voltage source of Emf = 20 kV. The nonlinear load is modeled by a 100 kVA 6-pulse industrial diode bridge, feeding an R//C load on the DC side and an inductance LAC-Load = 400 μH on the load AC side.

The ability of the shunt active filter to eliminate the harmonics generated by the nonlinear load is validated via simulations. In this simulation, SAF includes the LCL filter connected to the network through the voltage source inverter while the injected current is controlled, firstly, by SMC, then by C-HOSM, then by 2-SMC Twisting, and, finally, by 2-SMC super-twisting algorithms, driven by the PWM with 16 kHz switching frequency. Moreover, the reference current is identified via the advanced PLL-based generalized identification method. 

Table 1 gives the rating values of the studied system.

In Table 1, $S_{nom}$, Δ$P_{elec}$, Δ$P_{mag}$, $u_{cc}$, and $I_{oc}$ represent the rated apparent power, copper losses, iron and magnetic losses, short-circuit voltage, and open circuit current of the distribution power transformer, respectively.

The values of the LCL passive elements are calculated to impose a cut-off frequency of 1900 Hz in order to achieve a −55 dB attenuation rate of the switching frequency components. Moreover, the storage parameter values ($C_{dc},\;V_{dc}$) are calculated to provide a 2% ripple ratio of the DC voltage while ensuring a good dynamic performance of SAF [25].

### 5.1. Simulation Results: Simulink Environment

The simulations are carried out using the SMC, C-HOSM, 2-SMC Twisting, and Super-Twisting control algorithms. 

**Figure 5:**
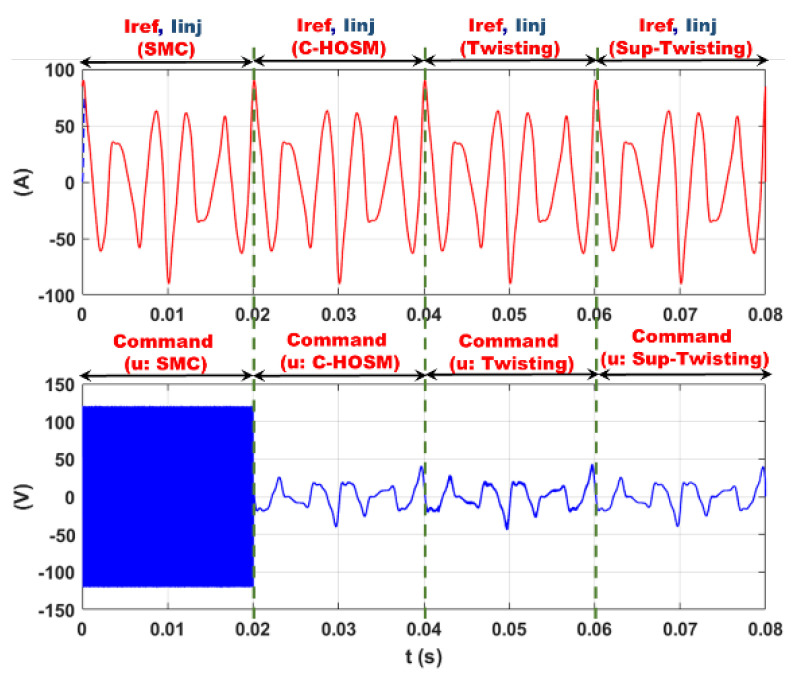
Tracking and controls for the SMC, C-HOSM, 2-SMC Twisting, and Super-Twisting algorithms.

A simple Simulink block scheme is studied here, where the current harmonics are modeled by harmonic current sources of ranks 5, 7, 11, 13, 17, 19, and 23 according to the case study in the second simulation set up. Figure 5 shows the simulation plots of $I_{ref}$, $I_{inj}$, and the control function u.

It can be observed that all four controllers ensure very accurate tracking with the SMC discontinuous control signal while the higher-order SMC controllers generate continuous control functions.

### 5.2. Simulation Results: Simscape-Electrical Environment

In the first simulation set up, the simulation is carried out using a comprehensive model of the Simscape-Electrical software, which includes a distorted network, characterized by total harmonic distortion of the PCC voltage equal to 4.45%. 

In these simulations, which are performed in either the time or frequency domains, the active filter is either activated from the beginning of the simulation or after six operation periods. The response time and the overshot are demonstrated in the two extreme cases.

**Figure 6:**
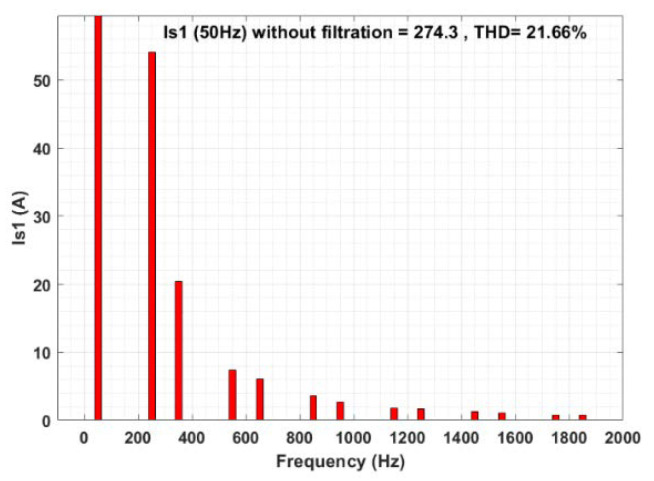
Spectrum analysis of network current before filtration.

The spectrum analysis of the load current is shown in Figure 6. From this figure, it can be observed that the current harmonics at the grid side $I_{s}$ before filtration have the conventional ranks: 5th, 7th, 11th, 13th, etc., while the amplitude value of the fundamental current is 274.3 A with a THD equal to 21.66%.

#### 5.2.1. SMC Controller

**Figure 7:**
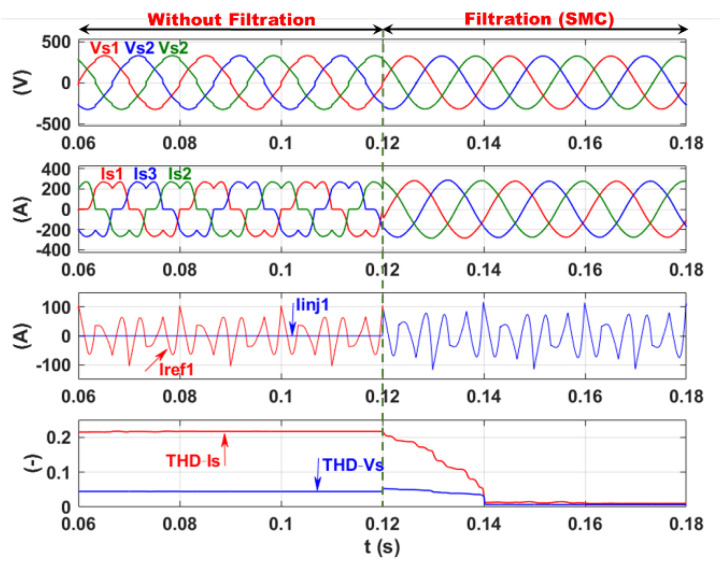
Current and voltage filtering using time domain SMC.

Figure 7 shows the simulation results of SAF, before and after filtering, respectively, for the three-phase network voltage ($V_{s123}$), three-phase grid currents ($I_{s123}$), identified harmonic current $I_{ref1}$ and injected current $I_{inj1},$ phase 1, superimposed, and total harmonic distortion of the current ($THD-I_{s}$) and voltage ($THD-V_{s}$) at the grid side superimposed, respectively.

From Figure 7, it is clear that the phase shift between the reference and the injected currents for high-frequency switching/discontinuous SMC disappeared even in the presence of the LCL filter. This ensures the sinusoidal line current and voltage patterns on the network side after filtering (at 0.12 s). This is reflected in Figure 7 by a significant decrease in both the current THD $\left(THD-I_{s}\right)\;$and voltage THD $\left(THD-V_{s}\right)\;$of phase 1 at the grid side from 21.6% and 4.45%, respectively, before filtering to 1.05% and 0.68%, respectively, after filtering. A good performance of the SMC within SAF is observed while the applicability of this structure of SAF is limited by the very high switching frequency/discontinuous control.

It is worth noting that the THD voltage is less than 1.6%, which is in line with the recommendations of Electricité de France (EDF) for specific applications [25]. Furthermore, in industrial zones, the THD voltage recommendations are limited by 5 to 8% (e.g., IEEE STD 519-2014) while the maximum THD current is limited by 20% for networks of $S_{sc}/S_{Load}>1000$, where $S_{sc}$ is the short-circuit power of the grid and $S_{Load}$ is the load power.

#### 5.2.2. C-HOSM Controller

**Figure 8:**
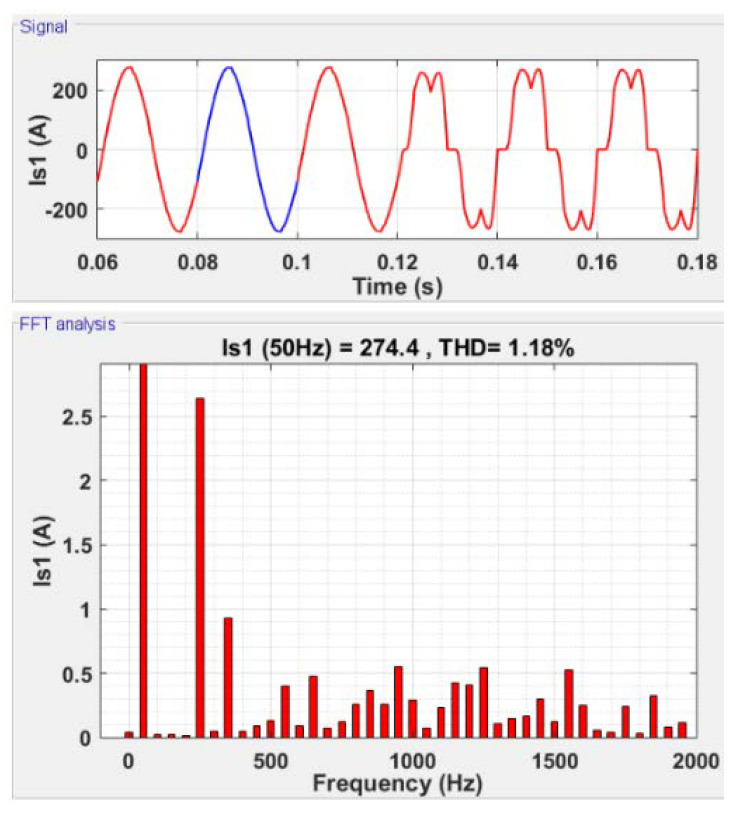
Current filtering using the C-HOSM controller.

In Figure 8, the time and frequency domain analyses are presented for the current at the network side using the C-HOSM controller. The active filter is, in this case, deactivated at 0.12 s. From Figure 8 (signal), an almost sinusoidal source line current on the network side with filtration can be observed. This is affirmed, as in Figure 8 (FFT analysis), by a large decrease in the current THD $\left(THD-I_{s}\right)\;$on the network side to 1.18% with filtration. This FFT signal can be compared with the FFT analysis of the same line current without filtration, as presented in Figure 6. 

On the other hand, the FFT analysis of the network voltage shows that the voltage THD $\left(THD-V_{s}\right)$ is reduced to 0.89% after filtration.

#### 5.2.3. 2-SMC Twisting Controller

**Figure 9:**
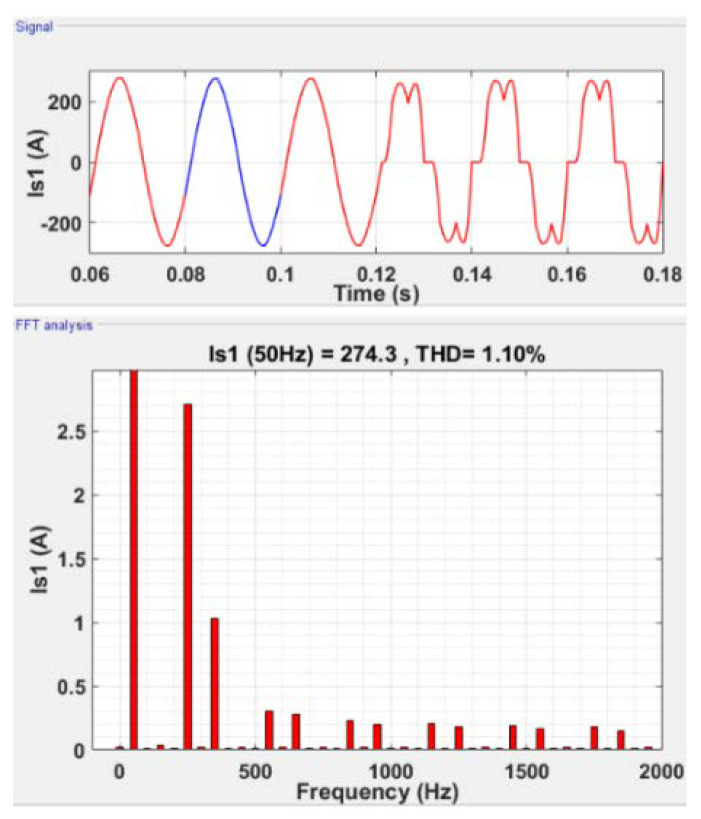
Current filtering using the twisting-controller.

The same simulations are performed using the 2-SMC Twisting controller, and the results are presented in Figure 9. In this case and from Figure 9 (signal), the sinusoidal line current pattern on the network side with filtration is caused by a large decrease in the current THD (THD-Is) on the network side to 1.10% (see Figure 9, FFT analysis). This occurs due to the decrease in the voltage THD (THD-Vs) to 0.66% with filtration.

#### 5.2.4. 2-SMC Super-Twisting Controller

**Figure 10:**
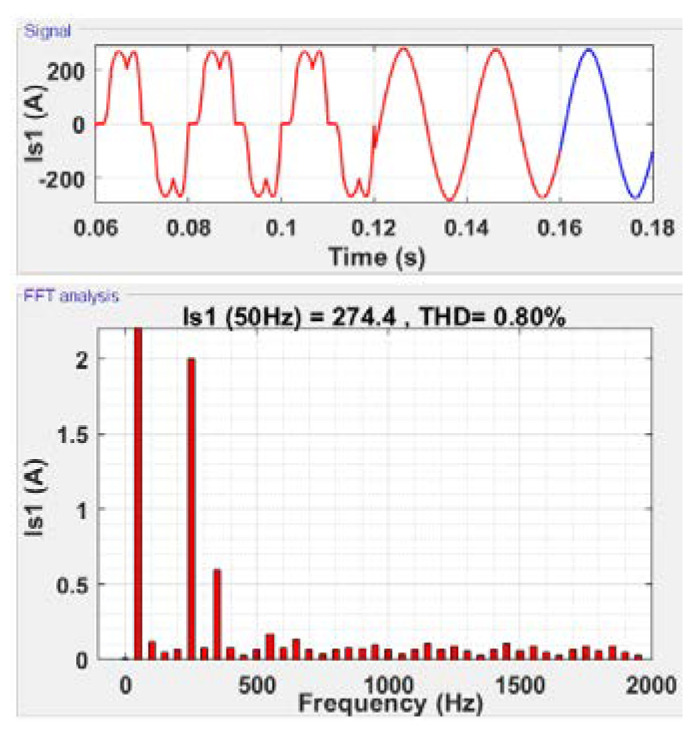
Current filtering using the super-twisting controller.

In the next set of simulation, the active filter operating with the 2-SMC super-twisting controller is activated after six operation periods. The results are presented in Figure 10.

From Figure 10 (signal), one can observe a sinusoidal shape of the line current on the network side after filtration. This is due to a significant decrease in the current THD $\left(THD-I_{s}\right)\;$on the network side to 0.8% as shown in Figure 10 (FFT analysis). The voltage THD $\left(THD-V_{s}\right)$ is reduced to 0.62 % with filtration.

### 5.2.5. DC Voltage and Energy Balance

In this subsection, SAF is controlled by the C-HOSM, Twisting, and Super-Twisting controllers to filtrate harmonics to 0.12 s and simulated. The compensation of the reactive power is achieved. 

**Figure 11:**
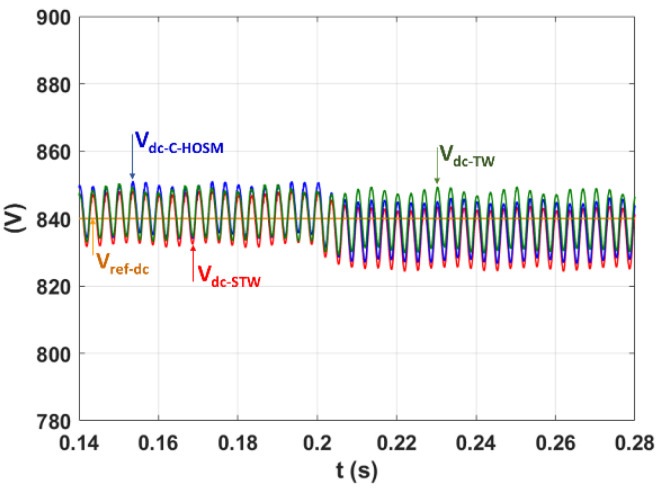
DC voltage using the C-HOSM, twisting, and super-twisting controllers.

Figure 11 shows the superimposed plots of the reference voltage $V_{ref-dc}=840\;V$ and the DC capacitor voltage $V_{dc}$ (see Figure 1) for the three proposed controllers. One can observe that the PI controller used for the DC bus voltage regulation ensures, within the three controller cases, almost the same good DC voltage tracking, where the fluctuation ratio is lower than the pre-calculated 2% based on Equation (7).

**Figure 12:**
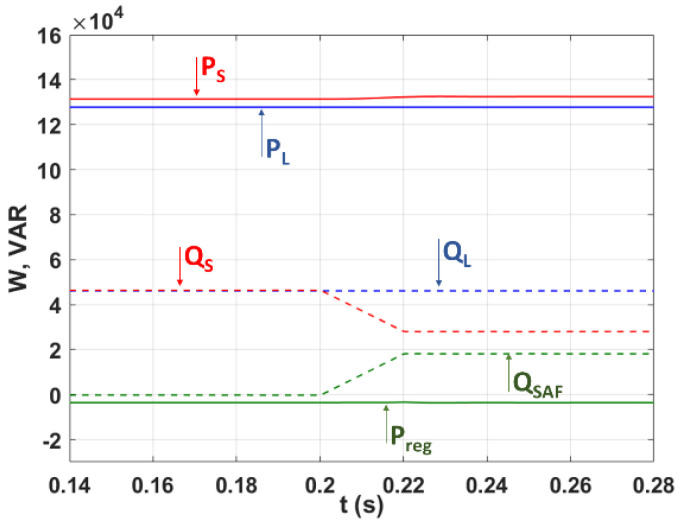
Energy balance of the SAF environment.

In the same context, Figure 12 presents:

(a) The power balance plots of the P_reg_ active power (see Figure 3 and Equation (6));
(b) The reactive power Q_SAF_ absorbed and provided by SAF; and
(c) P_S_ and Q_S_ delivered by the electrical network, and those consumed at the load side: P_L_ and Q_L_.

It is worth noting that in this simulation, the active filter compensates for only 40% of the global reactive power demand in order to avoid overloading of the active filter.

It can be observed from Figure 12 that it is obvious that throughout the simulation, the consumed/absorbed active and reactive powers are equal to the sum of those delivered by SAF and the grid. Furthermore, due to the advanced PLL, the DC bus charging power (P_reg_) using the advanced PLL is limited without additional reactive power consumption. Indeed, the advanced PLL simultaneously calculates the magnitude $V_{d}\;$and phase $\theta_{d}$ of (PSVC) $V_{d123}$.

## 6. Experimental Results

In this section, the real-time operation of the generalized instantaneous power identification algorithm that operates with the advanced PLL is tested and validated via an experimental study. 

### 6.1. Experimental Set Up

**Figure 13:**
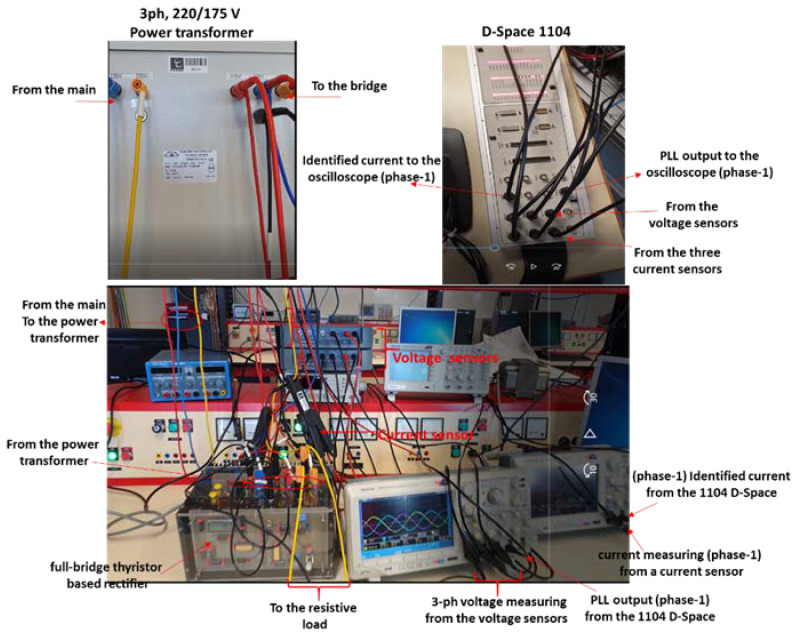
Experimental bench of the generalized identification method.

Figure 13 shows the experimental bench, consisting of a nonlinear load represented by a 3-phase 6-thyristor full-bridge rectifier, feeding a 4 kW/220 V variable resistive load. The full-bridge rectifier is based on STT116 thyristor-thyristor modules (116 A, 800 V), supplied successively by the 230/400 V 3-phase network, 3 kVA/(0–400 V) 3-phase autotransformer, and 3 kVA, (220/175 D/Yn) 3-phase power transformer. 

Additionally, the generalized identification algorithm that operates in concert with the advance PLL is implemented using the dSPACE CP1104 processor. Finally, the current and voltage measurements are performed by three current sensors and two differential voltage measuring devices.

### 6.2. Electrical Characteristics and Effects of Measuring Devices

In this experimental bench, the current and voltage are measured using a current sensor (E3N current clamp, type A) and a differential probe (MTX, type C), respectively. Both sensors are compatible with the MSO3014 oscilloscope shown in Figure 13.

The effect of the measuring devices on the control-command circuit of SAF and, consequently, on the filtering/compensation quality of SAF is studied and evaluated via a experimental study and then a real case study.

Indeed, in the real implementation, to obtain the same results as the simulation, the measurement devices, including the current and voltage sensors, should comply with very restrictive specifications. In fact, they must ensure high-accuracy measurement, neglecting the frequency domain gain and phase for the entire conventional bandwidth frequencies, a quick response (compared to the delay/sample time), a limited noise level, and a high rejection ratio of high-frequency disturbances. 

In this work, the frequency bandwidth of conventional harmonics is theoretically 2500 Hz (even if, in practice, it is less than 2 kHz), and the response time must be much less than the delay caused by the switching mode operation of SAF, which results in a delay in the injection of the current. Furthermore, the filtration/compensation currents are not applied instantly.

Thus, for the inverter block employed in the SAF modeling, the authors used the Universal Bridge of the Simscape Electrical toolbox. Indeed, the VSI model block takes into account a delay in the sampling period, and in the present case, the inverter switching frequency is set to equal 16 kHz. 

It is worth noting that the simulations are performed in the discrete time domain with a fixed step, with a sampling time of 1/16,000 ≈ 60 μs. The calculation is performed during this sampling period while the current is injected at the end of this period. 

Finally, Table 2 and Table 3 give the electrical characteristics of the measuring devices for the current and voltage. Thus, it is possible to evaluate their effects on the real-time results. Indeed, the accuracy, frequency response (in gain and phase), bandwidth frequency, disturbance rejection rate, response time, etc. of the measuring devices are decisive aspects of the power quality improvement.

#### 6.2.1. E3N-Type-A Current Sensor

This type of sensor is a current probe based on a hall effect cell and is intended for use with an oscilloscope (the MSO3014 oscilloscope in our case). Table 2 presents the electrical characteristics of this current measuring device.

#### 6.2.2. MTX-Type-C Voltage Differential Probe

MTX I032-C is a two-channel differential probe, which allows the differential input voltage to be attenuated and converted into a lower voltage, with two switches to select 1/10 or 1/100 as the attenuation coefficient. Table 3 presents the electrical characteristics of this voltage measuring device.

#### 6.2.3. Sensor Impact on the Reliability of the Experimental Results

From Table 1 and Table 2, it is clear that the characteristics of the current and voltage probes largely comply with the very restrictive specifications of SAF. Consequently, the experimental results are very close to the simulation results.

Indeed, the characteristics of the current and voltage measuring devices are as follows: the bandwidth frequencies of these devices are 100 kHz and 50 MHz, respectively; the rise and fall times are less than 4 μs and 7 ns; the noise levels are less or equal to 3 and 10 mV; and the accuracy is 3–4% and 3%, respectively. These characteristics fully comply with the required specifications (e.g., a 2 kHz bandwidth frequency, response time well below a 60 μs delay). Additionally, the current sensors’ frequency response guarantees the full detection of all harmonic signals, without gain and phase errors, which are 0 dB and 0°, respectively, for the entire bandwidth of the conventional harmonics. In the same context, the voltage probe provides almost complete rejection of high-frequency disturbances, e.g., −50 dB for 1 MHz.

### 6.3. Implementation Results

**Figure 14:**
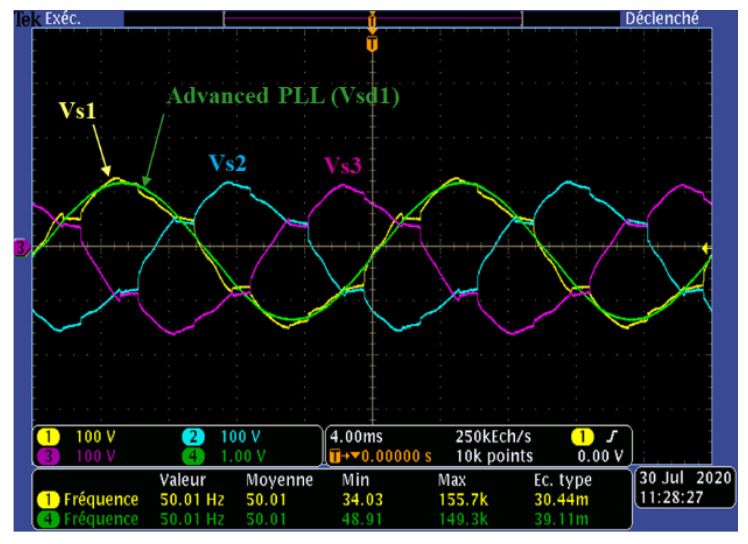
Experimental results of the three-phase grid voltage and positive sequence voltage.

**Figure 15:**
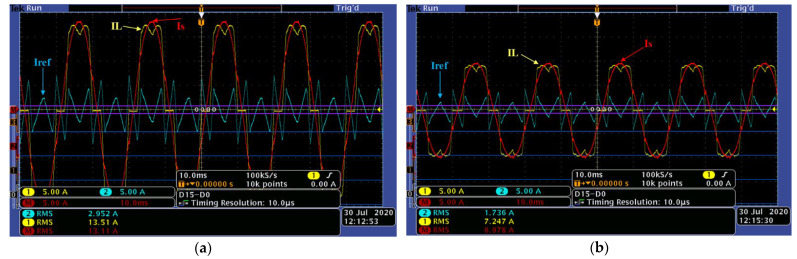
(**a**,**b**). Experimental results of the load current, distorted identified current, and grid current for two load levels (4 and 2.2 kW).

Figure 14 and Figure 15a,b demonstrate plots, which validate the effectiveness of the advanced PLL and the generalized identification method in highly distorted conditions.

In Figure 14, the grid side three-phase simple voltages (V_s123_) and the direct/positive voltage sequence of phase 1 (V_sd1_) delivered by the advanced PLL are presented. One can observe the high-quality performance of the advanced PLL. Indeed, despite the completely distorted form of the network voltage, the extraction of the positive sequence voltage, with a magnitude of 100 V, in phase with the load current (see Figure 14) at the same frequency as the fundamental grid voltage (50.01 Hz) testifies to the effectiveness, robustness, and accuracy of the advanced PLL.

On the other hand, Figure 15a,b shows, respectively, the two load levels (4 and 2.2 kW), phase 1, load currents IL (13.5 and 7.2 A), harmonic identified currents Iref (2.959 and 1.7 A), and grid side current Is (13.1 and 6.9 A). The completely sinusoidal form of the grid currents for the two load levels validates the high-quality performance of the generalized instantaneous power identification algorithm even under very distorted voltage conditions. It is worth noting that in this realization bench, the current Is is obtained by subtracting Iref from IL.

Finally, it should be noted that, after filtration, the sinusoidal shape of the grid side current proves the high accuracy in identifying the perturbation current caused by the nonlinear load, including extraction of the grid voltage positive sequence (in the amplitude and phase angle). This high level of precision in identifying a wide harmonic spectrum proves the reliability of the technical characteristics of the measuring devices, as presented in Table 2 and Table 3, and consequently proves the significant importance of the choice of the measuring devices.

## 7. Case Study

This case study offers an accurate modeling of the electrical network of a textile factory located in the industrial zone of Shaikh Najar in Aleppo city, Syria.

### 7.1. Specification of the Grid Site 

**Figure 16:**
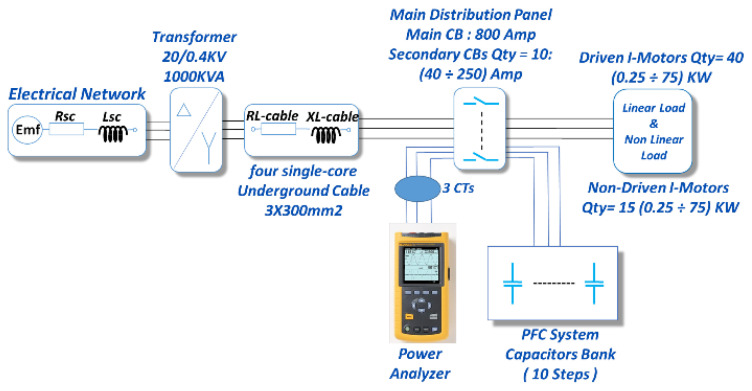
Electrical grid of the textile factory.

The distribution transformer of the considered textile factory (20 kV/0.4 kV) is powered by two overhead lines of 20 kV each, which come from the Shaikh Najar substation (66 kV/20 kV). This power transformer supplies the electric power to the site production line specifically from three-phase (two 300 mm^2^ conductors per phase) underground cables with 0.4 kV, as presented in Figure 16. 

The production process includes 40 driven and 26 non-driven asynchronous motors, with nominal powers ranging from 0.25 to 75 kW. The driven motors are controlled by cascaded (AC/DC/AC) converters. The power factor of the factory is 0.98 with capacitor-based PFC while the power factor is reduced to 0.76 when the power factor correction device is deactivated. The 282 kVA PFC bank consists of 10 steps of delta connection dispatched as follows: 2×50;2×40;2×25;2×16;2×10. The PFC device is installed on the side of the main distribution panel, which contains 10 circuit breakers of nominal current from 40 to 250 A, whereby all motors can be turned off or on depending on the production process, as shown in Figure 16.

**Figure 17:**
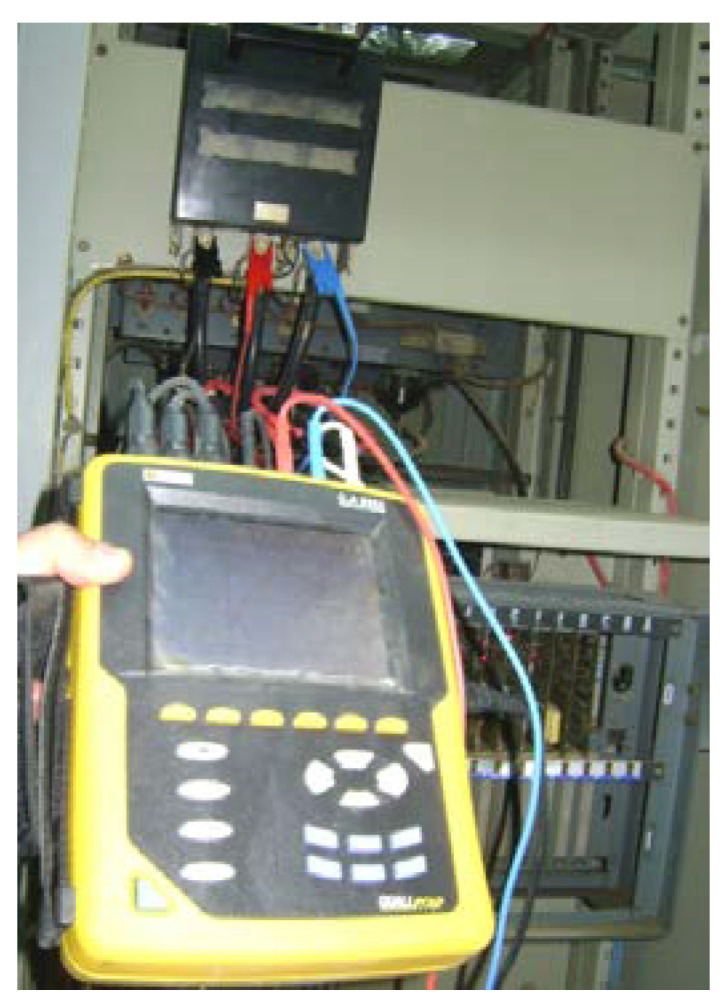
Power quality phenomena measurements using C.A 8334B.

Due to repetitive electrical malfunction impacting the factory productivity, many measurements were taken within a week using multiple C.A 8334B spectrum analyzers/power quality analyzers, as presented in Figure 16 and Figure 17.

### 7.2. Real Measurement Data of the Grid Site Using the Power Quality Analyzer Measuring Device

Since the modeling of such a complicated industrial network is based on the accuracy of real measurements, the electrical characteristics of the measuring devices have prime importance to ensure the construction of a reliable model that reflects the real impact of the electrical perturbations/power quality phenomena on the distribution networks.

Indeed, like in the previous real implementation, in order to obtain the same simulation results as those obtained via the real measurements, the measurement devices, e.g., power analyzer and current sensors, should comply with the specifications of the case study, with the objective of improving the power quality in the industrial electrical networks. 

The required specifications are high accuracy in the measurement of the electrical quantities, such as the fundamental current and voltage; active, reactive, and apparent powers; individual and total harmonic distortion of the current and voltage; current and voltage harmonics in amplitude and phase; power factor; unbalance ratio; fundamental frequency, etc.

In this real case study, the fundamental frequency is 50 Hz and the highest current harmonic rank is 50. In addition, the grid voltage is 400/230 V, the nominal load power ranges from 0.25 to 75 kW, the power factor varies, and the unbalanced current is 3%.

Finally, in this paragraph, the power quality analyzer specifications, including those of the current sensors, are detailed with respect to the case study objective, i.e., real modeling of an industrial site for power quality improvement.

#### 7.2.1. Electrical Specification of the C.A 8334B

The C.A 8334B is a three-phase power quality analyzer, which provides instant imaging of a grid’s principal characteristics and monitoring of their variations over a period of time. Its measuring sensors allow simultaneous management of all the measurement functions of the various magnitudes, phases, detection, continuous recording, etc. without any constraints.

The principal measurements carried out by the C.A 8334B are:

- Measurement of alternating (RMS) voltages up to 480 (phase/simple voltage) or 960 V (line/composite voltage) with uncertainty ±(5%+0.2 V);
- Measurement of alternating (RMS) currents up to 6500 A using sensors with an accuracy ± (0.5%+1 A); and
- Measurement of the frequency of 50 and 60 Hz networks with variations from 40 to 69 Hz with uncertainty ±0.01 Hz.

The main characteristics of the power quality analyzer device used in this case study to carry out the case study and to support the new methodology of modeling are presented in this paragraph. Indeed, all specifications relating to the current, voltage, harmonics, power factor, unbalance, etc. are specified in Table 4.

#### 7.2.2. Current Sensor Specifications

In addition to the specifications of the power quality analyzer, the characteristics of the current sensors are presented in Table 5.

In this context, the C.A 8334B power quality analyzer is designed to operate with the following current sensors: the MN probe, C probe, AmpFLEX (flexible three-phase current sensors), PAC probe, and adapter Box, with a secondary current value (5 or 1 A).

In this work, the C193 current sensor is adopted because of its adaptability to the case study constraints, in particular to the active filtering/compensation of the electrical perturbations. In this context, Table 5 shows the main sensor specifications.

### 7.3. Real Measurement Data of the Grid Site Using the Measuring Device with the Current Sensors

**Figure 18:**
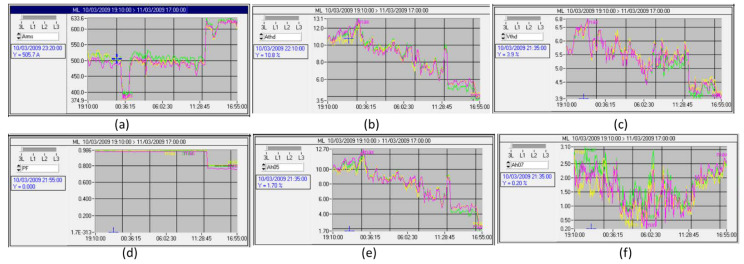
Real measurements of fundamental, harmonic currents, and voltage ratios and power factor using the power analyzer: (**a**) load current, (**b**) load current THD, (**c**) grid voltage THD, (**d**) power factor, (**e**) current harmonic distortion (rank 5), and (**f**) current harmonic distortion (rank 7).

Finally, the measurement plots, whose data was obtained by the QualyStar-View-Software of the C.A 8334B analyzer operating in concert with the C193 current sensors, during the period when the harmonic voltage distortion reaches its maximum from 3 October 2009 at 7:10 p.m to 3 November 2009 at 5 p.m, are shown in Figure 18.

This figure presents, respectively, at the distribution panel side the curves for the three phases (L1, L2, and L3) on (a) the effective values of load currents (in A), (b) the total harmonic distortion of the load current (in %), (c) the total harmonic distortion of the network voltage (in %), (d) the power factors, and (e and f) the individual load current harmonic ratios of ranks 5 and 7. It is worth noting that the spectrum analyzer was set to measure the first 50 harmonics (see [18] for more details).

### 7.4. Electrical Modelling of the Studied Site

The upstream network feeding the 20 kV/0.4 kV power transformer is modeled, as before, by the series connection of a voltage source of Emf = 20 kV and a short-circuit power impedance of Ssc = 500 MVA. The PFC device consists of the capacitor bank, activated and deactivated by controlled switches (see Figure 16).

Note that the models of the controlled harmonic loads are not presented in the Simscape Electrical Matlab toolbox; the only ones available in this toolbox are converters that drive induction or DC motors, etc. Therefore, it is very complicated to represent, in our real case study, the electrical network of such a site with 60 motors, 40 of which are driven by AC/DC/AC converters. Moreover, in order to simultaneously measure the currents and voltages in more than 40 nonlinear loads, a large a number of spectrum analyzers equal to the number of nonlinear loads are required.

Therefore, in this case study, nonlinear loads are modeled by their individual harmonic currents (provided by the measurement device) using current sources with parallel resistors with very high resistances [18,25].

This approach allows electric power systems of different industrial zones to be modeled and simulated independently of their size, type of load, and converter-based driving devices. Moreover, the number of power analyzers is equal to the number of production lines and not the number of nonlinear loads, as is commonly carried out. Finally, the rated values of the network, presented in Figure 16, are given in Table 6 and Table 1.

### 7.5. Modeling Validation and Evaluation of the Measuring Device Effects 

Based on Figure 18, the time (1:25 p.m/3 November 2009) represents the operating point when the total harmonic voltage distortion reaches a maximum. It is worth noting that in the industrial grids, the activation of a PFC device considerably increases both the total harmonic distortion of currents and voltages. The results are shown in Table 7 (see [18,25] for more details).

The site grid, presented in Figure 16, is modeled on the basis of the measurements delivered by the power quality analyzer (see Figure 16 and Figure 17 and Table 7), and the parameter values of the industrial factory network presented in Table 1 and Table 2. From the results of the real measurements and the simulation verification [18,25], it can be observed that all values are almost identical, with an accuracy of 97%. 

Here, $V_{s}$, $I_{L}$, $THD-V_{s}$, $THD-I_{L}$, $P_{Load}$, $Q_{Load}$, and $PF_{Load}$ represent the PCC voltage, load current with corresponding total harmonic distortions, overall active and reactive powers of the production line, and power factor, respectively.

This result confirms the reliability, robustness, and accuracy of the measuring devices and the new approach for modeling nonlinear loads in industrial areas. Therefore, all the simulation results, especially with the integration of SAF, are reliable.

Indeed, from Table 4 and Table 5, it is clear that the technical characteristics of the C.A 8334B power quality analyzer, including the C193 current sensors, represent typical measurement devices for the case study objective, i.e., improving the power quality in industrial electrical networks. Indeed, compared with the specifications of the case study in Section 7.2, the C.A 8334 allows measurement, with a very high accuracy level, of the fundamental frequency from 40 to 69 Hz, grid voltage up to 960 V, nominal apparent power (depending on the current sensor) up to 9999 kVA, power factor from −1 to 1, unbalanced current up to 100%, harmonic rank up to 50 with THD, and individual harmonic ratios 99.9%, etc. In the same context, the C193 current sensor complies with the characteristics of the power analyzer with almost the same precision, e.g., a nominal current up to 1200 A, fundamental frequency from 48 to 65 Hz, harmonic components’ measuring accuracy of 1% up to 1 kHz, then 2% for harmonic frequencies > 1 kHz, etc.

It is worth noting that errors caused by the spectrum leakage phenomenon could deteriorate the THD spectrum analysis. This occurs when the duration of the measuring window is not the total multiplicity of the fundamental period of the measured signal. These errors can cause changes in the values and displacements of higher-order harmonics. In addition, the components of the sub-harmonics and inter-harmonics could appear [38].

In order to reduce the effects of the spectral leakage phenomenon on the calculation accuracy of the harmonic bins, the C.A 8334B power quality analyzer, used in this paper, applies the FFT (16 bits) 1024 samples on 4 cycles without windowing, which complies with the recommendation of the CEI 1000-4-7 norm [39].

Finally, based on the real measurement with the new modeling approach and experimental implementation, taking into account the accuracy of the used measuring devices and sensors, the following simulation results incorporating SAF are reliable. 

### 7.6. Simscap-Electrical Environment with Shunt Active Filter

SAF operates, in this second simulation set up, within a highly distorted network, characterized by a THD voltage at the PCC, equal to 6.8%, with a current unbalance factor of 3% (see Figure 18a and Table 7). In this simulation set, the SAF is activated after 5 periods of 20 ms, operating firstly with the C-HOSM controller, then with the 2-SMC Twisting, and finally with the 2-SMC Super-Twisting controller.

#### 7.6.1. C-HOSM Controller

**Figure 19:**
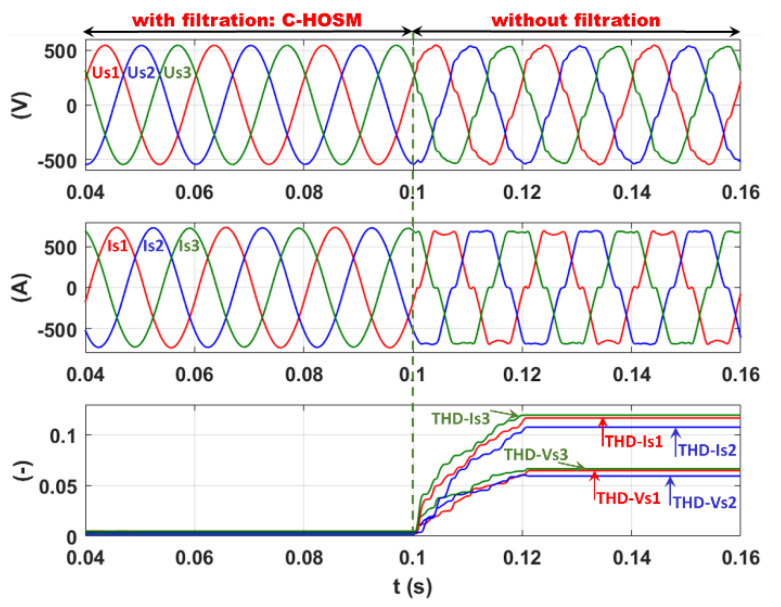
Current filtration: C-HOSM controller.

In Figure 19, the simulation results for SAF are presented with and without filtration for the three-phase network composed of voltages ($U_{s123}$), line grid side currents ($I_{s123}$), total harmonic distortion of current ($THD-I_{s}$), and voltage ($THD-V_{s}$), respectively, using the C-HOSM controller are demonstrated. From Figure 19, one can observe the sinusoidal shape of the current and voltage at the grid side with filtration. This is reflected by the 0.59%, 0.55%, and 0.66% voltage THD at PCC for phases 1, 2, and 3 with filtration while it is 6.7%, 6.2%, and 6.8% without filtration (see Table 7). This important decrease in the voltage THD is caused by a significant decrease in the current$\;THD\left(THD-I_{s}\right)$ from 11.6%, 10.7%, and 12% (see Table 7) without filtration for the three phases to 0.92%, 0.65%, and 0.75% with filtration.

#### 7.6.2. 2-SMC Twisting Controller

**Figure 20:**
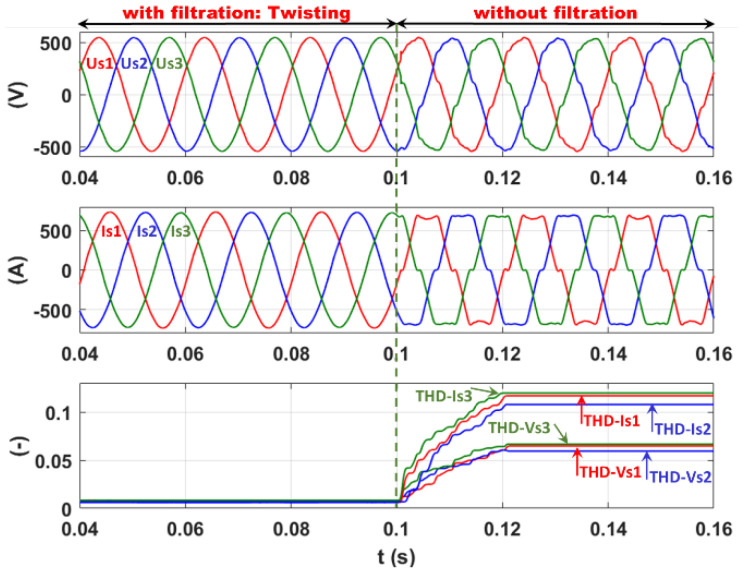
Current filtration: 2-SMC Twisting controller.

The same simulation sequence is performed using the 2-SMC Twisting controller, as shown in Figure 20, where a large decrease in the current THD to 0.55, 0.35, and 0.42% for phases 1, 2, and 3, respectively, leads to a significant reduction in the voltage THD to 0.23, 0.16, and 0.25%. This result confirms the very high-quality level of filtration ensured by the 2-SMC Twisting controller.

#### 7.6.3. 2-SMC Super-Twisting Controller

**Figure 21:**
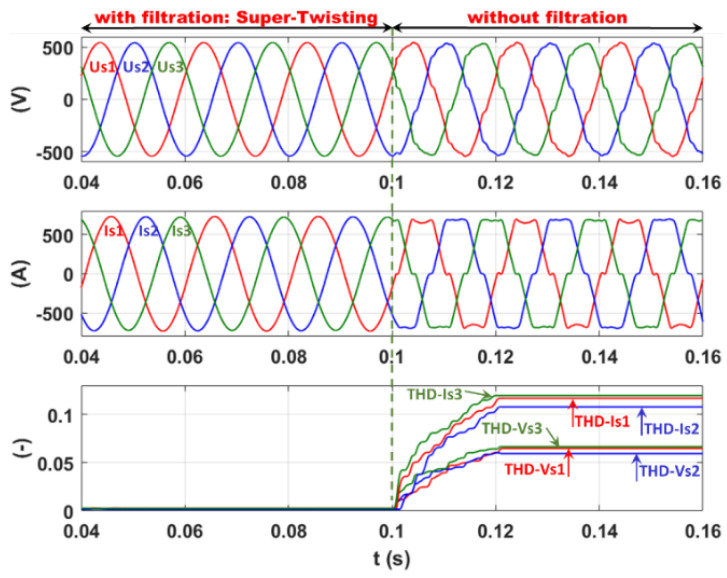
Current filtration: 2-SMC Super-Twisting controller.

Finally, the 2-SMC Super-Twisting controller is validated within the same highly distorted real network and following the same simulation sequences as before. The corresponding plots are presented in Figure 21.

From Figure 21, the perfect sinusoidal form of both the currents and voltages at the network side confirms the excellent performance of this control method. This high level of filtration quality is shown via a significant decrease in the current THD to 0.31%, 0.15%, and 0.23% and, consequently, the voltage THD to 0.2%, 0.15%, and 0.22%, respectively, for phases 1, 2, and 3.

Therefore, all three proposed control algorithms ensure robust continuous control signals and a very good filtering quality. In addition, the THD voltage is well below the 1.6% required by most constrained recommendation standards (EDF norm). This very important result is reached despite the unbalanced current and, consequently, the voltage, significant voltage harmonic distortion of the factory electrical grid, and the presence of the LCL filter.

## 8. Conclusions

The effects of measuring devices/sensors on improving the power quality of electric networks were studied in this paper. It was shown that the characteristics of measuring devices have a significant impact on the real implementation and real measurement/simulation of SAF and, consequently, on the power quality improvement in electrical networks. Indeed, the technical characteristics of the measuring devices (i.e., the C.A 8334B power quality analyzer, including the C193 current sensors, and the E3N current sensors associated with the MTX voltage differential probes) complied with the required specifications in this research, which resulted in accurate and reliable results.

In this context, a structure of the LCL/SAF control system based on second- and higher-order sliding mode control (2-SMC/HOSMC) was proposed in a recently published patent [25] and studied in this work in detail.

Various features of the proposed structure show its superiority with respect to the existing ones. Firstly, the proposed LCL-2SMC/HOSMC/SAF structure is a modern alternative to the conventional L-series/SAF structure, with a light, small, economical, and easy to size LCL output filter compared to the series-inductance one. Indeed, the sum of the LCL inductances (200 μH in the present case) are 10–20 times (or more) less than the L-series inductance of 2–5 mH. Furthermore, it allows higher attenuation of the switching-frequency components. Secondly, the phase lag phenomena, introduced by the linear controllers, and the very-high-frequency switching impact, caused by the classical SMC, are avoided by the use of the proposed 2-SMC/HOSMC controllers with a consecutive PWM, which guarantees the required switching frequency of the control function. In this case, no overheating of the inverter devices is expected. Thirdly, the generalized instantaneous power identification method, using the proposed advanced PLL, is still effective, unlike the traditional method, even under a severely perturbed network. In addition, the advanced PLL, extracting both the phase and magnitude of the positive sequence PCC voltage, and the DC bus charging current is limited without additional reactive power consumption. It is worth noting that the conventional PLL, with only phase detection, results in additional active power consumption from the grid.

The experimental results validate the generalized identification method, which includes the advanced PLL even under highly distorted network voltage. In addition, current and voltage measuring devices (the E3N current sensor and the MTX voltage differential probe, compatible with the MSO 3014 oscilloscope) were chosen to ensure reliable and accurate experimental results.

In this work, the linear (RST: roots locus), SMC, and continuous C-HOSM and 2-SMC Twisting and Super-Twisting algorithms were tested via simulations. The simulation results, obtained using MATLAB, Simulink, and Simscape-Electrical code, demonstrated the accurate and robust filtering quality of SAF, controlled by the proposed control algorithms, for the industrial nonlinear load and the practical case study under a distorted and significantly distorted voltage network (4.5% and 6.8% with a current unbalance factor of 3%). In the case study, the production line of the textile factory was modeled based on real measurements provided by the C.A 8334B power quality analyzer and simulated using QualyStar View Software. The real measurements of the individual harmonic currents support the accurate modeling of the driven motors. The incorporation of this modeling approach, which was validated with almost a 97% accuracy, facilitates the simulation of industrial zones regardless of their rated power, nonlinear load type, and driving device type. The C.A 8334B measuring device, including the C193 current sensors, in the presented analysis considers the effects of measuring devices on the performance of the real industrial networks.

## 9. Patents

The results of this study partially validate the recently published patent: “Device for Active Electrical Compensation”. US Patent US20220200282A1, filed 2 July 2019, and issued 23 June 2022.

## Figures and Tables

**Table 1:** Rated values of the electrical grid.

**Electrical Network and Power Transformer**	
Emf, Ssc, Rsc, Lsc	20 kV, 0.5 GVA, 250 mΩ, 2.4 mH
S_nom_, ΔP_elec_, ΔP_mag_, u_cc_, I_oc_	1 MVA,1.05%, 0.17%, 6 %, 1.3 %
**Shunt Active Filter**	
LCL coupling filter	L_fi_ = 100 μH, L_fg_ = 100 μH, R_fi_, R_fg_ = 5 mΩ, C_f_ = 130 μF
Energy storing element	C_dc_ = 0.6 mF, V_dc_ = 840 V
LCL Cut-off frequency	1900 Hz
Switching Frequency	16 kHz

**Table 2:** Electrical characteristics of the E3N current clamp.

Range	Measurement Range	Uncertainty	Pass Band	Noise Level	Rise and Fall Time	Loaded Impedance	Frequency Response	
							Gain	Phase
100 mV/A	50 mA to 10 A peak	3%	100 kHz	840 μV peak-peak	<4μs	1 MΩ//100 PF	to 2 kHz: 0 dB ± 3% 10 kHz: −3 dB	to 2 kHz: 0° 10 kHz: −12°
10 mV/A	50 mA to 40 A peak	4%		3 mV peak-peak				

**Table 3:** Electrical characteristics of the MTX differential probe.

Range	Measurement Input	Main Power Supply	Uncertainty	Pass Band	Noise Level	Rise and Fall Time	Impedance	Common Mode Rejection
1/100	±600 V	230 VAC ± 10%, 50/60 Hz	3%	50 MHz	10 mV dc	7 ns	1 MΩ//13 PF	80 dB at 50 Hz 50 dB at 1 MHz
1/10	±50 V							

**Table 4:** Electrical specifications of the power quality analyzer measuring device.

Measurement		DC Voltage (V)	DC Currents (A) (PAC Probe Sensor)	Active Powers (kW)	Reactive Powers (kVAR)	Power Factor	Unbalance	Phase Angle	THD &Harmonic Ratios Rang ∈ [1; 50]	Harmonic Angles
Measuring Range	Min	6	1	0	0	1	0%	−179°	0%	−179°
	Max	680	1700	9999	9999	−1	100%	180°	999%	180°
**Uncertainty**		±(1% + 0.5 V)	±(1% + 1 A)	±(1.5%) max	±(1.5%) max	±(1.5%) Cos ϕ ≥ 0.5	±(1%)	±(2°)	±(1%+0.5%)	±(3°) rang ∈ [1; 26]
						±(1.5%+0.01) 0.2≤ Cos ϕ <0.5				±(10°) rang∈ [26; 50]

**Table 5:** Electrical characteristics of the C193 current sensor.

**Nominal range**		1000 A AC for f ≤ 1 kHz		
**Measurement range**		3 A to 1200 A AC (I > 1000 A not permanent)		
**Input/Output ratio**		1 mV AC/A AC		
**Maximum clamping capacity**		52 mm		
**Frequency & Distortion factor in the reference conditions**		48–65 Hz & < 1% without superimposed DC current		
**Precision in the reference conditions**	**Rang (A)**	3–10	10–100	100–1200
	**Amplitude**	≤0.8%	≤0.3%	≤0.2%
	**Phase Shift**	≤1°	≤0.5°	≤0.3°
	**Frequency**	30–48 Hz: <0.5%	65–1 kHz: <1%	1–5 kHz: <2%
**Variations in the nominal field of use (to be added to the error under reference conditions)**				
**Frequency in relation to accuracy**		30–48 Hz: <0.5% 65–1000 Hz: <1% 1–5 kHz: <2%		
**Distortion of crest factor ** **≤** ** 6** ** and current ** ** ≤ 3000 A peak**		<1%		
Distortion DC current ≤ 15 A < 1% superimposed on the nominal current		<1%		
**Overload: in relation to frequency (beyond 1 kHz)**		Imax ≤ 1000 × 1/f (kHz)		

**Table 6:** Catalogue rating values of the industrial site cable.

Electrical Cable	
2 × (3 × 300) mm^2^, 20 m long	XL-cable = 255 mH/km, RL-cable = 29.5 mΩ/km

**Table 7:** Electrical network quantities.

DATE and Time	3 November 2009	1:25 p.m	49.98 Hz
Phases123	L1	L2	L3
V_s123_ (V)	224.5	223.8	224.2
I_L123_ (A)	522.19	518.3	507.1
THD-V_s123_ (%)	6, 7	6, 2	6, 8
THD-I_L123_ (%)	11,6	10,7	12
P_L123_ (kW)	114,379	114,054	110,071
Q_L123_ (kVAR)	18,812	15,860	24,532
PF_Load123_	0, 97	0, 98	0, 96

## Data Availability

Not applicable.

## Nomenclature

$C_{dc}$	DC bus capacitor	F
$I_{inj}$	Injected current	A
$I_{ref}$	Identified current	A
$L_{f}$	Inductance of the output filter	H
$L_{g}$	Grid equivalent inductance	H
$\tilde{P}$	Perturbing real power	VA.a
$\tilde{Q}$	Perturbing imaginary power	VA.r
$\bar{Q}$	Reactive power	VAR
$R_{f}$	Internal resistance of the output filter	Ω
$R_{g}$	Grid equivalent resistance	Ω
$S_{Load}$	load apparent power	VA
$S_{n}$	Rated apparent power of the power transformer	VA
$S_{sc}$	Short-circuit power of the grid	VA
$u_{kk}$	Short-circuit voltage of the power transformer	%
$V_{d123}$	Three-phase direct voltage components	V
$V_{d}$	Direct voltage component magnitude	V
$V_{dc}$	DC bus voltage	V
$V_{inv}$	Inverter output voltage	V
$V_{s}$	Point of common coupling Voltage	V
$\Delta P_{copper}$	Copper losses of the power transformer	W
$\Delta P_{oc}$	Iron and magnetic losses of the power transformer	W
$\theta_{d}$	Direct voltage component angle	°
**Abbreviations**		
C-HOSMC	Continuous higher order sliding mode controller	
MPPT	Maximum Power Point Tracking	
PLL	Phase Locked Loop	
PSVC	Positive Sequence Voltage Components	
PQ	Power Quality	
PWM	Pulse-width modulation	
SAF	Shunt active filter	
SMC	Sliding mode controller	
VSI	voltage source inverter	
